# Synergistic effects of organic carbon and silica in preserving structural stability of drying soils

**DOI:** 10.1038/s41598-024-58916-9

**Published:** 2024-04-09

**Authors:** Luis Alfredo Pires Barbosa, Mathias Stein, Horst H. Gerke, Jörg Schaller

**Affiliations:** Research Area 1 “Landscape Functioning”, Working Group “Silicon Biogeochemistry”, Leibniz Centre for Agricultural Landscape Research (ZALF), Eberswalder Strasse 84, 15374 Müncheberg, Germany

**Keywords:** Environmental sciences, Hydrology

## Abstract

Predicted climate warming and prolonged droughts pose a threat to the soil structure as organic carbon losses weaken the stability of soil aggregates. Well-structured soils are important for storage and movement of water, solutes, and air, the development of plant roots, as habitat for soil organisms, and the microbial activity. Structural stability is measured in terms of hydro-mechanical properties. This study compares effects of amorphous silica with those of organic carbon on stability parameters during drying of aggregates from relatively finer- and coarser-textured soils. Silica amendment enhanced the positive effect of organic carbon on structural stability in terms of the tensile strength. Synergistic effects between silica and organic carbon in soil colloids appear to dynamically alter aggregate density and friability (i.e., ability to crumble) during drying. Silica together with organic carbon could help soil management to reduce negative effects of predicted prolonged droughts on soil structure and stability.

## Introduction

Global warming and intensive land use significantly increase the risk of the soil's porous network collapsing, particularly as organic carbon losses^1–3^ weaken the wet and dry stability of soil aggregates^4^. Consequently, in the face of extreme precipitation and prolonged droughts, the soil structure, delineated by pores and the spatial arrangement of solids^5^, faces a heightened risk of degradation^6^. This degradation has negative impacts on soil environmental and agricultural functions, since the reduced aggregate structural stability leads to the collapse of soil pore network, hampering water retention and gas flow, nutrient dynamics and root development^7^. Additionally, it negatively affects microbial activity^3,8^, ultimately undermining soil stress tolerance and carbon storage^9,10^. Given the need to achieve a 43% reduction in greenhouse gas emissions by 2030^11^ while ensuring agricultural production, it is crucial to explore methods and mechanisms to enhance soil resilience and restore degraded soils amid challenging climatic events^12^. One example is the amendment of inorganic materials (e.g. amorphous silica) that has been shown to enhance water storage in drying soils^13,14^. The suggested mechanism involves enhancing bonding among soil particles upon drying^15^, caused by meniscus forces and stiffness of organic carbon, and by dehydration-induced contraction in silicates. The surface tension of the water draws the soil particles into closer proximity as it evaporates. The points where the particles touch dry last, concentrating any dissolved substances, like silica and organic carbon, at these contact points^16^. However, the impact of silica on soil aggregate stability and mechanisms of amorphous silica interactions with mineral particles and soil organic carbon remains widely unknown mainly because of a lack of experimental evidence.

Organic carbon enhances the stability of the solid particles arrangement in soil by associating with minerals, strengthening the interparticle binding forces in soil^17^. This fosters the formation of micro-aggregates within macro-aggregates^18,19^, enhancing mechanical stability (i.e. aggregate tensile strength) of the soil structure under wet and dry conditions^4^. These microscopic forces encompass physicochemical and biological processes that influence soil agricultural and environmental functioning such as pore architecture, biological activity, and nutrient recycling across various scales. Assessing these microscopic binding forces can be efficiently and cost-effectively achieved through macroscopic measurements, such as friability^20^, which quantifies aggregate tensile strength and reflects the pore scale differences between micro and macroaggregates^21^. Additionally, there is an inverse relationship between aggregate volume and density, which has often been described in terms of the fractal dimension^22^. Higher friability values and lower fractal dimension values are quantitative physical properties that indicate a relatively good quality of soil structure^20^, referring to a well-developed pore connectivity and stable arrangement of solids^23^, and thus, signifying that the soil structure is effectively supporting its environmental and agricultural functions^7^. A better soil structure is promoting long-term protection of soil organic carbon^10^ and enhancing, among others, the hydraulic conductivity^24^ and oxygen flow for microbiome functions^3,8^, determining soil fertility.

However, intensive soil management^25^ and rising global temperatures^2^ contribute to soil carbon depletion, making the soil structure more vulnerable during heavy rainfall events and prolonged droughts, thus increasing the risk of erosion^6^ by water and wind. In arable soils, the lack of structural stability leads to increased susceptibility to fragmentation and compaction caused by agricultural machinery and tillage practices^26,27^, resulting in the collapse of pore network, measured by lower friability values^28^ and increased fractal dimension^29^, a phenomenon known as structural homogenization^23^. These detrimental processes diminish the soil capacity to store organic carbon^30^, triggering a chain of reactions of structural soil degradation. Although the soil structure affects local functions response (e.g. water retention and microbial activity), it also brings implications for global-scale climate^31^. Nevertheless, amidst the extensive focus on organic carbon, it is noteworthy that inorganic materials such as amorphous silica may also act as binding agents in structured soils, particularly, in the context of the drying process^16^.

**Figure 1:**
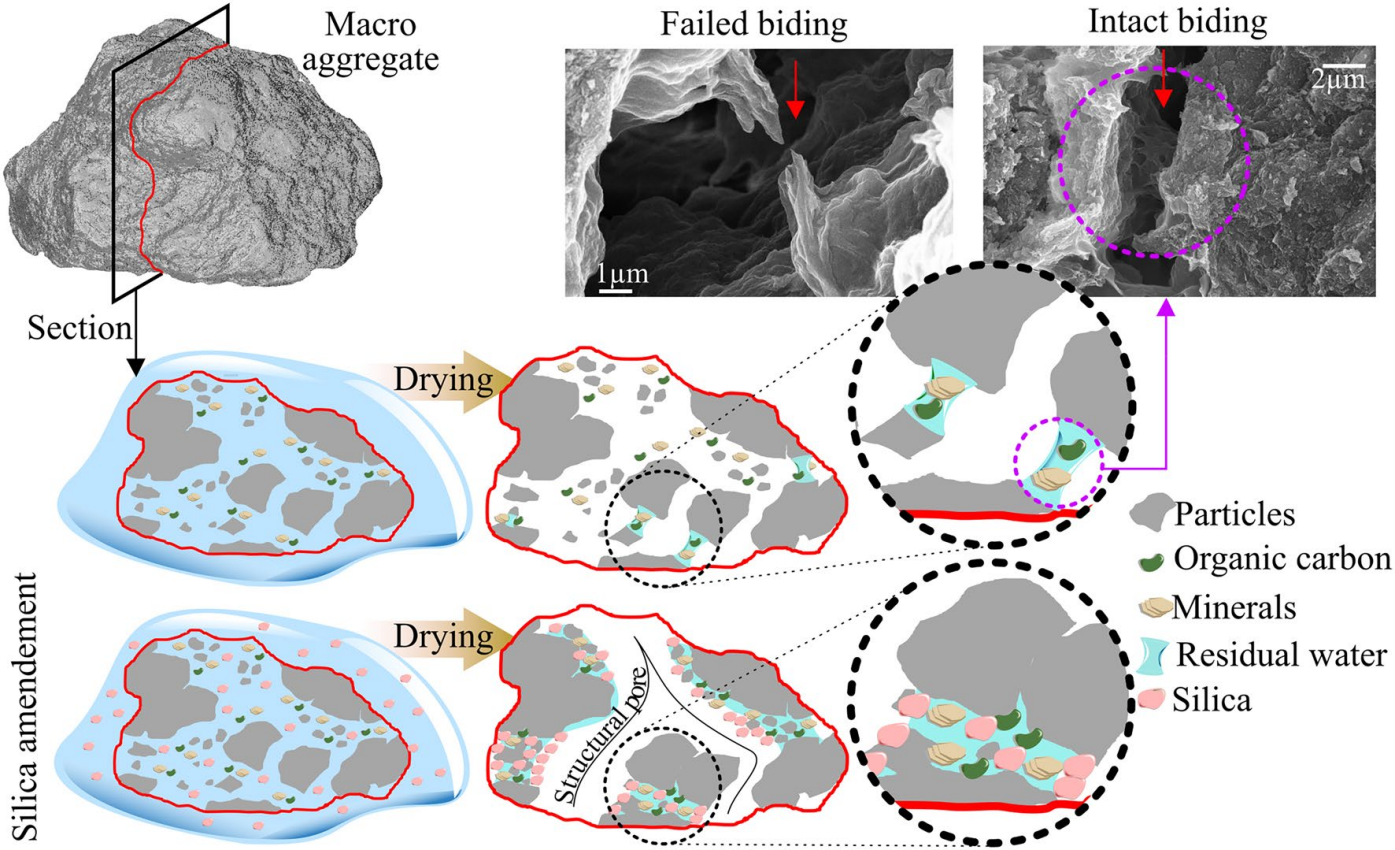
Illustration of aggregate cross-section showcasing the influence of organic carbon and silica on structural dynamics and binding stabilization throughout the drying process. Electron microscopy images depict both the intact and disrupted clay-organic colloidal material binding between soil particles of untreated soil.

Amorphous silica (later referred to as silica) naturally originates from litter fall as it precipitates from silicic acid in the plant or precipitates from mobilized silicic acid when soil dries^32^. This material possesses a high specific surface area and a high porosity, it may fill larger pores and bind or “aggregate” smaller particles. Furthermore, a silica layer may be created on the surface of soil aggregates^33^, thus enhancing the binding area for connecting particles^34^ (Fig. 1) and positively influencing the inter-particle binding forces^35^. For these reasons, silica is suggested to promote soil aggregation and increase structural stability^15,36,37^, as described by Uehara et al.^16^. Another crucial property of silica is its ability to shrink upon drying and swell upon wetting^38^, causing dynamic changes to the pore structure. These dynamics significantly increase soil water retention and improve hydro-structural stability^39^. The latter is indirectly enhancing soil water holding capacity and crop water availability^13,14^. However, specific effects of silica on the stabilization of soil aggregates and silica interactions with soil organic carbon are not fully understood^32^.

This study determines the importance of silica amendment, organic carbon, texture, and the combined effects of silica and organic carbon in relation to water content, soil stability, and density under drying conditions. This was studied for three aggregate size classes of two arable soils. Our hypotheses were that (i) soil organic carbon has a predominant effect on structural stability, (ii) silica amendment can further enhance aggregate stability, and (iii) synergistic effects of organic carbon and silica are observed especially under drying conditions. These effects help preventing the structured soil from homogenization and concurrently increasing soil resilience.

## Results

### Organic carbon and silica contents of the aggregate samples

The organic carbon content of the coarser-textured Endogleyic Colluvic Regosol was found to be significantly higher (*p* < 0.05) as compared to that of the finer-textured Haplic Luvisol (Table 1 and supplementary material SF.1). For the Haplic Luvisol samples, all aggregate sizes exhibited approximately 0.5% organic carbon content, and no statistically significant differences (*p* < 0.05) between treatments were observed. For the samples from the Endogleyic Colluvic Regosol soil, the organic carbon values were slightly above 1%, with no differences within the control and silica treatments (*p* < 0.05). The inorganic carbon content ranged from 0.01 to 0.04% across all aggregate sizes and treatments in both soils (Table 1 and supplementary material SF.2). For the smallest aggregate size in the silica-amended Endogleyic Colluvic Regosol, the inorganic carbon content was significantly higher (*p* < 0.05) as compared to that of aggregates of size classes 2–5 mm and 7–13 mm in the Haplic Luvisol control, as well as in the 2–5 mm aggregates of the silica-amended Haplic Luvisol. The silica amendment significantly increased (*p* < 0.05) the total silica concentration from 0.75% of both control soils to approximately 1.6% for all aggregates (supplementary material SF.3). However, no statistical differences (*p* < 0.05) were observed when comparing aggregate size classes within treatments and soils.

**Table 1:** The total organic carbon (TOC [%]) and total inorganic carbon (TIC [%]) for the soils and treatments utilized in the study, along with the percentage of amorphous silica [mg mg^−1^] and their respective standard deviations (SD).

		Aggregate size [mm]	TOC	SD	TIC	SD	Silica [mg mg^−1^]	SD
Haplic Luvisol	Control	2–5	0.5525	0.0378	0.0133	0.0010	0.7688	0.0759
		5–7	0.5182	0.0305	0.0148	0.0030	0.7105	0.0559
		7–13	0.5417	0.0552	0.0145	0.0017	0.7150	0.0362
	Silica-amended	2–5	0.6050	0.0088	0.0135	0.0006	1.6608	0.2480
		5–7	0.5690	0.0712	0.0175	0.0090	1.8283	0.0289
		7–13	0.5823	0.1204	0.0155	0.0051	1.5264	0.3614
Endogleyic Colluvic Regosol	Control	2–5	1.2172	0.0622	0.0253	0.0025	0.9517	0.0323
		5–7	1.1420	0.0813	0.0228	0.0022	0.8735	0.0195
		7–13	1.0953	0.0869	0.0213	0.0028	0.7978	0.0958
	Silica-amended	2–5	1.1738	0.0774	0.0365	0.0284	1.6164	0.2166
		5–7	1.1255	0.0542	0.0225	0.0019	1.6516	0.0964
		7–13	1.1827	0.0881	0.0223	0.0015	1.5749	0.0282

### Controlling factors for the hydro-mechanical soil properties

**Figure 2:**
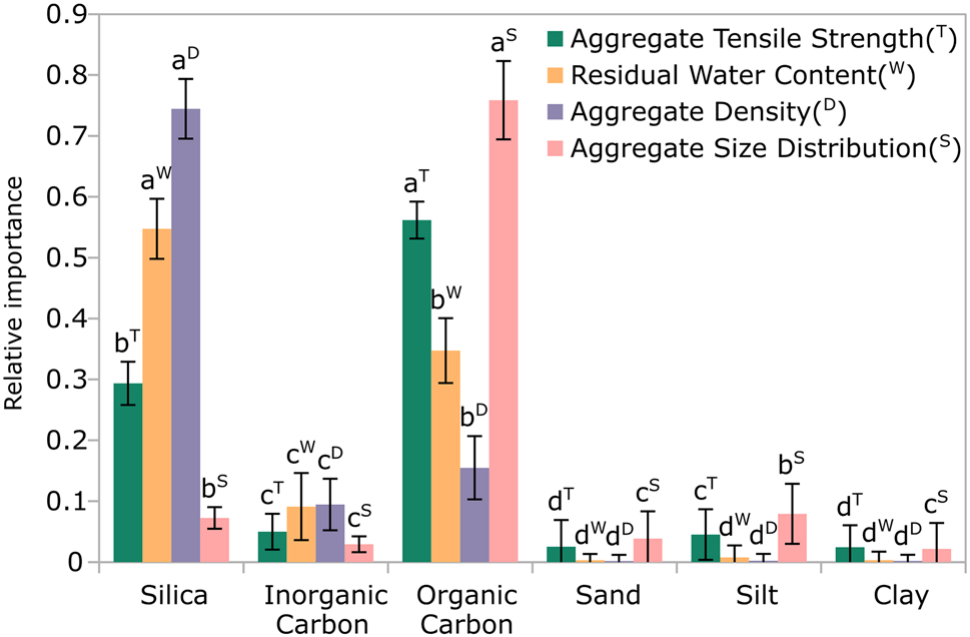
Relative importance of each factor for explaining physical aggregate properties obtained from the trained random forest algorithm. Letters indicate significant differences within each physical property (two-way ANOVA with Tukey’s post hoc test, *p* < 0.05). Note that an optimal configuration for the multiple output regression Random Forest (RF) model was achieved with 200 estimators, a maximum depth of 100, and a random state of 5. The overall score of the model was 0.73, and the mean absolute percentage error was 11% for tensile strength, 15% for water content, and 6.5% for density.

Organic carbon was found the foremost influential factor in exhibiting a positive impact on aggregate tensile strength and playing a pivotal role in increasing the aggregate size (Fig. 2). However, the silica amendment was found to be the primary factor for increasing both aggregate density and residual water content (Fig. 2). This is supported by the results obtained with the SHAP algorithm (SHapley Additive exPlanations) explaining the trained random forest algorithm (supplementary material SF.4). While variations existed among the importance of inorganic carbon, sand, silt, and clay for explaining aggregate properties, their significance was lower as compared to the more substantial effects of silica and organic carbon.

### Tensile strength and friability

**Figure 3:**
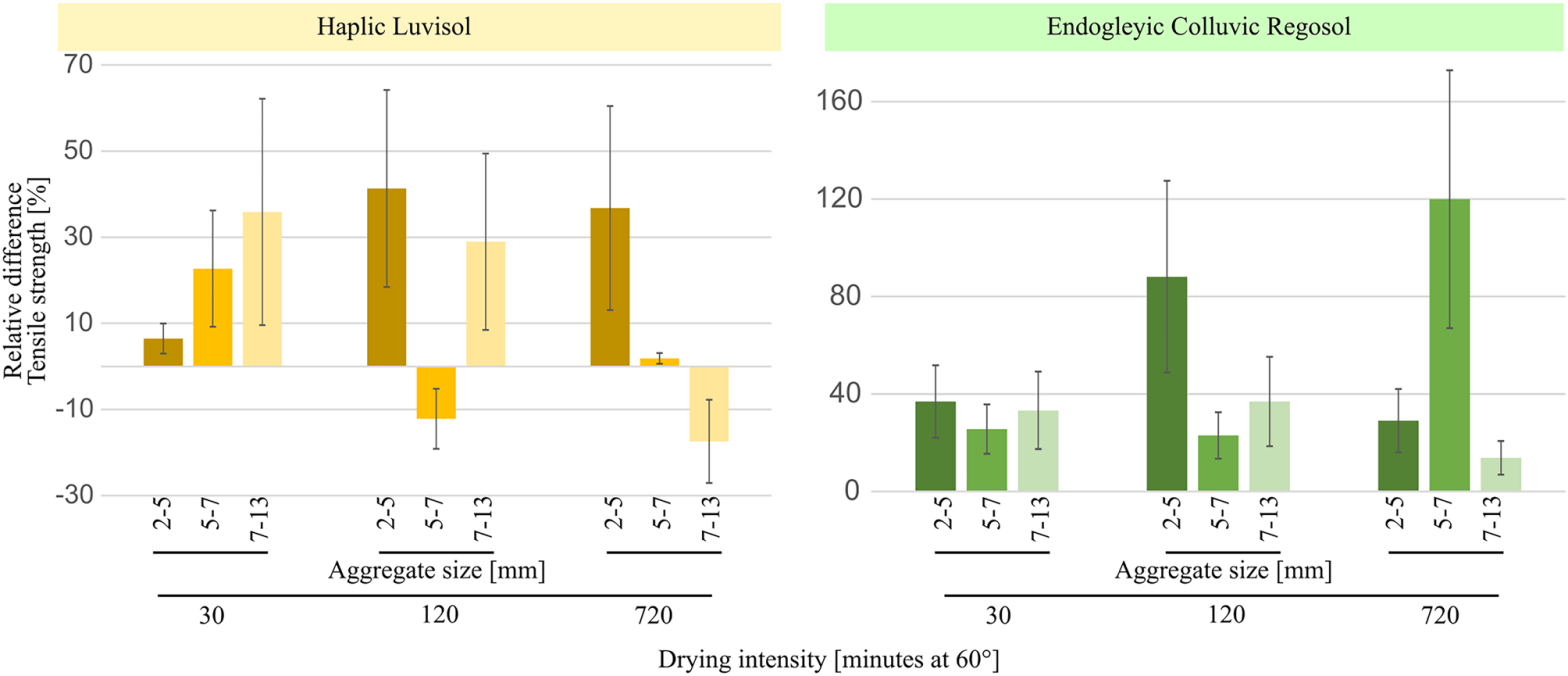
Relative difference between the aggregate tensile strength of samples from silica amendment plot and that of the control as a function of aggregate size and drying intensity for both soils. Vertical lines indicate the relative standard deviation.

For Endogleyic Colluvic Regosol, silica addition increased the aggregate tensile strength and counteracted the effect of the drying intensity (Fig. 3). While the addition of silica to the Haplic Luvisol increased the tensile strength of up to 40% compared to the control (Fig. 3), the overall tensile strength was not significantly affected by both silica amendment and drying intensity (supplementary material SF.5). However, increasing the drying intensity, the scale effect of tensile strength across different aggregate sizes of the Haplic Luvisol, as quantified by the friability, decreased significantly for the control treatment (supplementary material SF.6). In the case of the Endogleyic Colluvic Regosol, the addition of silica yielded a beneficial impact on aggregate tensile strength, especially noticeable in smaller aggregates (Fig. 3). Furthermore, increasing the drying duration (i.e. drying intensity) substantially magnified the gap in tensile strength between the smallest and largest aggregates, as shown by an increase in the friability value (supplementary material SF.6), providing evidence of structural aggregation. This could be because smaller aggregates tend to strengthen when drying, while larger ones tend to weaken, possibly due to the effect of widening of inter-aggregate pores between smaller aggregates. The control and silica amendment treatments of the Endogleyic Colluvic Regosol showed considerably higher tensile strength values compared to both the control and the silica amended Haplic Luvisol, particularly for the size class 2–5 mm (supplementary material SF.5). For the Endogleyic Colluvic Regosol, the silica amendment had a significant effect on the tensile strength of aggregates in the size class 2–5 mm (supplementary material SF.5), increasing it by 37, 88 and 29% when compared to the control treatment for the drying intensities of 30, 120, and 720 min, respectively (Fig. 3). Increasing drying intensity for the Haplic Luvisol control noticeably reduced the differences of the tensile strength between the smallest and largest aggregate, thereby reducing friability values (supplementary material SF.6), reflecting the structural degradation.

**Figure 4:**
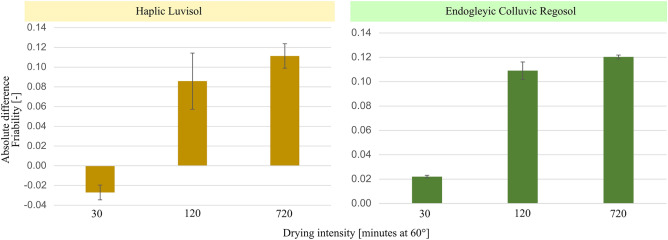
Absolute difference in friability between values calculated from silica amendment and those from control samples (i.e., amended ‘minus’ control) as a function of the drying intensity for both soils. Vertical lines indicate the relative standard deviation.

The addition of silica helped to maintain a more stable aggregate structure, which is manifested by increasing differences in the friability values as compared to the control treatment with the drying intensity for samples from both soils (Fig. 4). Silica led to an approximate fivefold increase in friability values with drying durations from 30 to 120 min (Fig. 4).

### Residual water content

**Figure 5:**
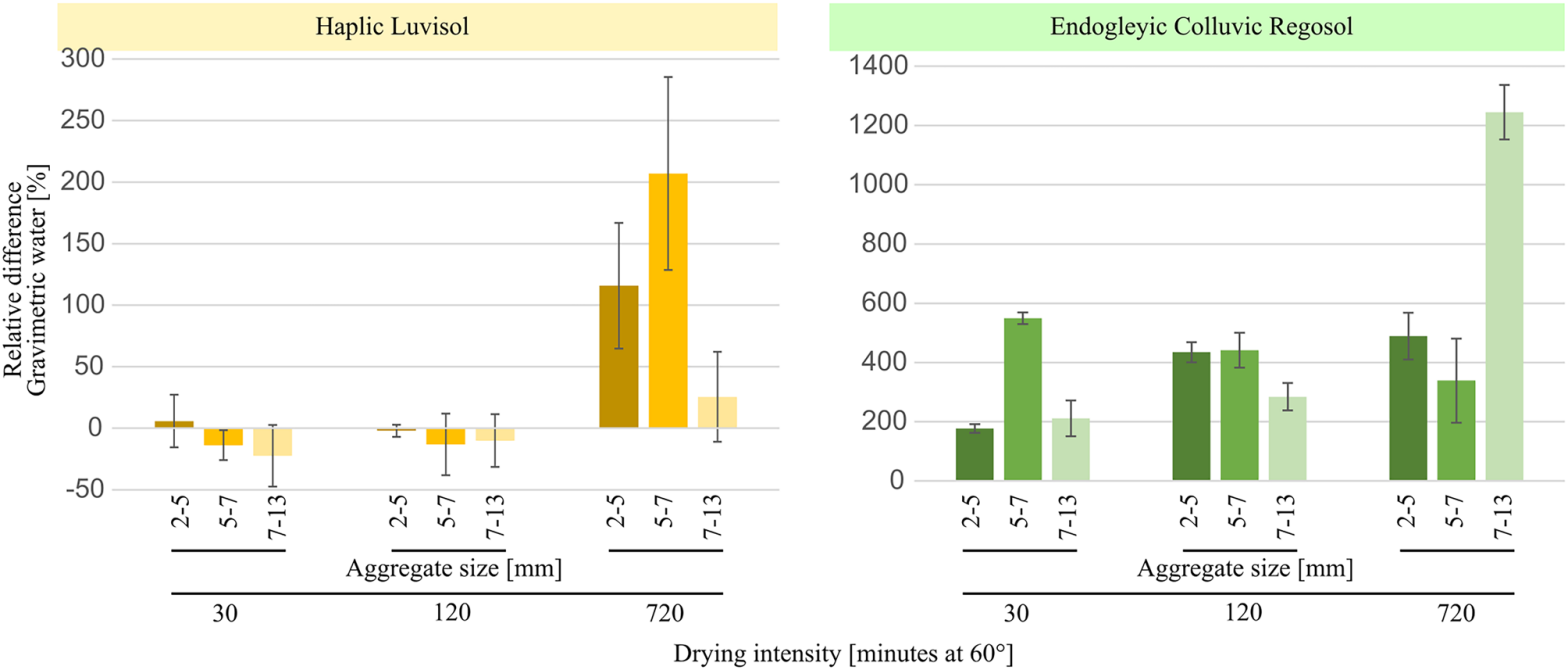
Relative difference in gravimetric water content between silica-amended and control samples, correlated with aggregate size and drying intensity for both soil types. Vertical lines indicate the relative standard deviation.

Silica addition to the Endogleyic Colluvic Regosol significantly increased the residual water content for all aggregate sizes across all drying intensities as compared to the control (Fig. 5). The exception was the 7–13 mm aggregate size class at drying intensity of 720 min, which was not significant despite the larger relative difference (Fig. 5). For the Haplic Luvisol, the silica amendment only led to increasing water contents at highest drying intensity (Fig. 5). Furthermore, at drying intensities of 30 and 120 min, the addition of silica to the Endogleyic Colluvic Regosol resulted in larger remaining residual water content as compared to that of the Haplic Luvisol (Fig. 5). During severe drought events, forage grasses (Lolium perenne) have been observed to extract water at matric potential of − 10 MPa^40^, resulting in 0.09 cm^3^ cm^−3^ for Haplic Luvisol and 0.07 cm^3^ cm^−3^ for Endogleyic Colluvic Regosol, as predicted from fitted retention curves^41^. However, the water content in the smallest aggregates from silica-amended Endogleyic Colluvic Regosol ranged from 0.04 to 0.01 cm^3^ cm^−3^ for drying intensities of 30 and 720 min, respectively. A water content of 0.04 cm^3^ cm^−3^ for the Endogleyic Colluvic Regosol possibly corresponds to a matric potential of 100 MPa, a phenomenon that can occur naturally, as air-dry aggregates can reach 100 MPa^42^.

Smaller aggregates retained more water than larger ones upon longer drying (supplementary material SF.7). However, silica did not affect residual water content of the Haplic Luvisol (supplementary material SF.7). The gravimetric water content measured for the control samples of the Haplic Luvisol at the first drying intensity was 0.015 g g^−1^, 0.011 g g^−1^ and 0.004 g g^−1^ for the 2–5, 5–7 and 7–13 mm aggregate size classes, respectively. The prolongation of drying from 30 to 120 min did not lead to a significant reduction in water content. However, the 720 min drying caused the most significant drop in water content to values of 0.002 g g^−1^, 0.001 g g^−1^, and 0.0006 g g^−1^, for the 2–5, 5–7 and 7–13 mm aggregate size classes, respectively, for the control samples and 0.004 g g^−1^, 0.003 g g^−1^, and 0.0008 g g^−1^, for the silica-amended treatment (supplementary material SF.7).

For the Endogleyic Colluvic Regosol, the aggregate samples from the control at all drying intensities resulted in significantly lower water contents as compared to those from the Haplic Luvisol (supplementary material SF.7). The Endogleyic Colluvic Regosol control exhibited higher susceptibility to water loss upon drying as compared to the Haplic Luvisol.

### Aggregate size distribution

**Figure 6:**
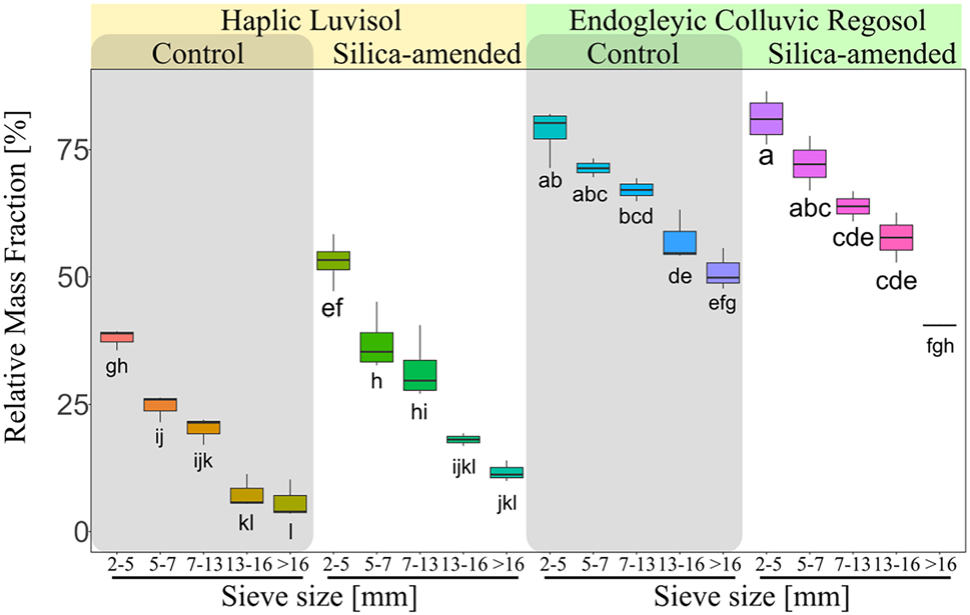
Box plot of cumulative aggregate size distribution obtained from hand-sieved size fractions. Letters indicate significant differences (Two-way ANOVA with Tukey’s post hoc test, *p* < 0.05). Note that the gap towards 100% relative mass is closed by the fraction 0–2 mm (not displayed).

The Endogleyic Colluvic Regosol displayed higher mass of aggregates across all size fractions considered when compared to the Haplic Luvisol (Fig. 6). Furthermore, the linear regression analysis (not shown) revealed that applying silica led to an increase in the slope (absolute value) of the aggregate size distributions. This means that the mass of smaller aggregates increased more compared to larger ones. Specifically, for the Endogleyic Colluvic Regosol, the slope rose from 1.89 to 2.58, and for the Haplic Luvisol, it increased from 2.21 to 2.77 (R value > 0.94).For Endogleyic Colluvic Regosol, a significant 37% increase in mass was observed in the 2–5 mm size class, while an even more pronounced 53% increase in mass of the 5–7 mm size class of the Haplic Luvisol (Fig. 6).

### Aggregate density and fractal dimension

Density values exhibited no significant differences among size classes, soil types, and remained unaffected by silica (supplementary material SF.8). However, silica addition caused a 5% increase in the density of the smallest aggregate size of the Endogleyic Colluvic Regosol. Although the increase in density for the smallest aggregate size fraction after the addition of silica was not significant, it led to a significant reduction of 0.5% in the fractal dimension of the silica-amended Endogleyic Colluvic Regosol. This reduction points to an enhancement in the density scale effect across different aggregate sizes (supplementary material SF.9). Conversely, no differences in the fractal dimension were observed for the control of both soils and the silica-amended Haplic Luvisol samples.

## Discussion

Our results outline a scenario in which organic carbon and silica emerge as prominent factors in controlling the physical properties of soil aggregates. While organic carbon plays a crucial role in stabilizing the aggregates and increasing the size, silica demonstrates its relevance in enhancing the residual water content and the aggregate density. As a result, the increased organic carbon content (supplementary material SF.1) combined with silica amendment significantly enhanced the mechanical stability of aggregates from silica-amended Endogleyic Colluvic Regosol (supplementary material SF. 5). This improved soil structure quality, as evidenced by an increase in friability value (supplementary material SF. 6) during the drying process.

The major importance of organic carbon to predict aggregate tensile strength (Fig. 2) corroborates its role in strengthening the binding forces among soil particles^19,43^ and enhancing soil structural aggregation^23^ (Fig. 6). Such enhanced aggregation is known to improve the connectivity of pores and increase meso- and macroporosity contributing to the increment of hydraulic conductivity^39^. This is known to be an indirect effect of organic carbon on residual water^44^, which is reflected the lower water content observed in the aggregates of the control treatment of the Endogleyic Colluvial Regosol at lower drying intensities (i.e., 30 and 120 min; as shown in Fig. 5). The permeation of silica into these structural pores notably improved the residual water content of the Endogleyic Colluvic Regosol (Fig. 5). This enhanced residual water content is attributed to the water-holding capabilities of silica itself^13,14^. Moreover, smaller aggregates retained more water at all drying intensities due to the presence of smaller pores according to the hierarchical structural formation principle^18^. Note that after drying at 60 °C, the residual water is adsorbed to solid surfaces and hold at water potentials > pF 6^45^ (> 100 MPa).

The application of silica notably increased the density of the 2–5 mm aggregate size within the Endogleyic Colluvic Regosol (supplementary material SF.8), which led to a considerably smaller fractal dimension (supplementary material SF.9). A decrease in fractal dimension is an indication of improved soil aggregation^22,46^, which may be explained by the flexibility of the silica chains that enables substantial shrinkage^38^ without breaking the contacts^34^. The presence of such characteristics, along with the elevated levels of clay and organic carbon in Endogleyic Colluvic Regosol, might aid in the aggregation of soil colloids by silica during the shrinkage process upon drying. The mechanism of silica rearrangement or deposition processes^16,32,34^ during the shrinkage and that of silica combined with soil colloids could have increased the number and size of bridges between particles^35^. This mechanism is additionally underpinned by the results of the importance analysis (Fig. 2), which emphasized the pronounced significance of silica in its positive influence on aggregate density (supplementary material SF.8). Although the density measurements did not reveal significant differences (supplementary material SF.8), the fractal values validate the improvement in structural quality (supplementary material SF.9).Particle binding forces have a direct and positive effect on aggregate mechanical stability (i.e., tensile strength)^35^ and organic carbon is the main binding agent controlling it (Fig. 2). This effect is particularly pronounced in smaller aggregates (supplementary material SF.5), which is in agreement with previous studies^20^, showing that the smaller the aggregate pores, the more sensitive the tensile strength is to particle binding forces^47^. Thus, the effect induced by silica on the aggregate structure during the drying process increased the effect of organic carbon on the tensile strength of the aggregates by strengthening the interparticle binding forces. Therefore, the silica amendment in the Endogleyic Colluvic Regosol together with organic carbon, led to the most substantial increase in tensile strength observed in the 2–5 mm size class aggregates (supplementary material SF.5). While rising the drying intensity, the difference in tensile strength between the smallest and largest aggregate increased, leading to an increase in friability as the Endogleyic Colluvic Regosol dried (supplementary material SF.6). This quantification suggests that the interplay of silica, organic carbon, and content of finer mineral particles enhanced structural aggregation of the Endogleyic Colluvic Regosol during the drying process.

In contrast, the control treatment of the Haplic Luvisol demonstrated increased vulnerability to soil structural collapse with higher drying intensity (supplementary material SF.6). However, the addition of silica improved the structural resilience of the Haplic Luvisol to drying, effectively minimizing structural deterioration at elevated drying intensities, as indicated by higher friability values (supplementary material SF.6). This showcases the stabilizing capability of silica on the structure of Haplic Luvisol during the drying process. Since friability has been shown to be driven by the length of intra-aggregate pores^20^, the increase in friability observed in our study (supplementary material SF.6) suggests the elongation of pore (or crack) length across different aggregate sizes upon drying. This can be attributed to the collapse of the pore network and the formation or expansion of pre-existing cracks during shrinkage. The latter occurs when the shrinkage of plasma porosity^48^ is greater than the bulk soil deformation^49^. As a result, the silica shrinkage pulling soil colloids together during the drying process as discussed above, may reduce the plasma porosity while enlarging pre-existing cracks as the soil dries.

The shrinkage of silica during the drying process may dynamically alter the soil water retention curve due to changes in the physical properties (e.g. fractal dimension and friability), and models for predicting hydraulic properties should consider a non-rigid pore structure^50^. The trade-off between pore collapse and formation affects the soil shrinkage curve, leading to the development of cracks, widening of structural pores, or closure of pre-existing macropores^49,51^. Therefore, studies should be conducted on undisturbed samples of silica-amended soils to quantify water retention combined with structural shrinkage, and models should be adapted to better explain this mechanism. Furthermore, the increased stability provided by silica mitigating the structural collapse in the soil caused by the drying process needs to be investigated at field scales. This is because the stability provided by silica, can prevent or reverse excessive soil structural breakdown processes (e.g., freezing and thawing, tillage), which is important for reducing vulnerability of structured soils to water and wind erosion^6^. Additionally, silica can reduce the turnover rate of aggregates^9^, which is important for stabilization mechanisms of soil organic carbon and consequently carbon sequestration^30^.

In summary, this study comprehensively examined the intricate effect of silica and soil organic carbon for two soil textures on the resilience of the density and mechanical stability of differently-sized aggregates. Silica amendment to the relatively finer-textured Haplic Luvisol soil was found to enhance structural aggregation and to further intensify the effectiveness of organic carbon on the mechanical stabilization of aggregates, protecting structural degradation upon drying. For the relatively coarser-textured Endogleyic Colluvic Regosol soil, the silica amendment was even more effective in improving the mechanical stabilization of aggregates. The findings reveal important synergistic effects of silica together with soil organic carbon concerning soil structure and stability.

## Material and methods

### Soils and sample preparation

The experimental field for soil collection is situated in the Uckermark region, northeastern Germany (53°23′ N, 13° 47′ E). This area experiences an average annual precipitation of 489 mm and an annual mean air temperature of 8.6 °C, as observed at the Dedelow Experimental Field Station of the Leibniz Centre for Agricultural Landscape Research (ZALF), Müncheberg. Two field treatments were considered for this investigation, control and amorphous silica amendment (silica). Silica was applied as Aerosil 300 (Evonik Industries, Germany) to the soil by first mixing the powder to 800 wt. % of water and then applying the mixture to the soil surface. After drying, a rotary hoe was used to mix the first 15 cm soil layer. Soil samples were collected 30 weeks after silica application from 4 plots of each treatment. Minimally disturbed soil cubic samples (0.1 m × 0.1 m × 0.1 m) were collected using a rectangular shovel from a depth of 0.05 to − 0.10 m of two different soils. They were classified according to FAO classification scheme WRB (IUSS, 2007) as Haplic Luvisol and Endogleyic Colluvic Regosol and represent soils of a typical catena of this landscape. The sand, silt and clay contents were 586, 321 and 93 g kg^−1^ for the Haplic Luvisol and 614, 208 and 105 g kg^−1^ for the Endogleyic Colluvic Regosol^41^.

The soil samples were transferred to the laboratory, placed on trays, and dried at ambient conditions. Subsequently, the clods, in the following denoted as soil aggregates, were separated along the pre-existing visible cracks^52^.

### Aggregate size distribution

Air-dried soil samples were weighed and then gently hand-sieved to isolate distinct aggregate size fractions: < 2 mm, 2–5 mm, 5–7 mm, 7–13 mm, 13–16 mm, and > 16 mm, after field treatments. Mechanical shaking was deliberately omitted to prevent any potential damage to the aggregates. Sieving was continued until no further material could pass through the sieve. Subsequently, the fractions were weighed, and the cumulative percent retained of each size fraction was calculated.

### Tensile strength and soil friability

For the tensile strength measurements, three size classes from the smallest fraction were used, 2–5 mm, 5–7 mm, 7–13 mm. This choice was based on the fact that the tensile strength of aggregates in the smallest size fraction is more sensitive to detect changes resulting from alterations in management practices^20,53^.

Each size class of aggregates was placed in cylinders measuring 5 cm in height and 5 cm in diameter. These cylinders were subsequently positioned in a sandbox to saturate them from the bottom by capillary action for a duration of three days. After the saturation period, the pressure head was adjusted to approximately 5 kPa by lowering the outflow level. This drainage process led to an increased stability of the aggregates for manual handling. Thus, 15 aggregates were randomly collected^20^ from each of the 3 size classes (i.e., 15 × 3), from each block (15 × 3 × 4), soil texture (15 × 3 × 4 × 2) and treatment (15 × 3 × 4 × 2 × 2) and for three water (15 × 3 × 4 × 2 × 2 × 3) content levels (i.e., a total of 2160 variants) and placed in petri dishes. During the selection process, aggregates without visible stones were chosen. After aggregates were collected from saturation, the water level was determined by different drying intensities using an oven set at 60 °C for 30 min, 120 min, and 720 min, to simulate hot weather conditions at bare top soil. After the samples were removed from the oven, they were placed in a desiccator for cooling without humidity absorption before the tensile strength measurements were carried out.

The tensile strength was measured (on a total of 2160 samples) using the indirect tension test^54^ with the Tension Testing Machine^55^. This test consists basically of a controlled aggregate rupture while the forces are monitored. The point of failure for each aggregate was detected when a sudden drop in force^56^. The rupture force, $F_{n} \left[ N \right]$, was used to calculate the tensile strength, $\sigma \left[ {Pa} \right]$^54^:

1 $$\sigma = 0.576\frac{{F_{n} }}{{d^{2} }}$$

where $d \left[ m \right]$ is the diameter of an individual aggregate, calculated as^57^.

2 $$d = \left( {\frac{6w}{{\pi \rho }}} \right)^{\frac{1}{3}}$$

where $w \left[ g \right]$ is the mass of the individual aggregate and $\rho \left[ {{\text{g}}\;{\text{ cm}}^{ - 3} } \right]$ is the average bulk density for aggregates within the same size class.

The values of $\sigma$ of the fifteen aggregates from each petri dish were then used in a probability distribution function to determine the probability of aggregate failure. The Weibull model, a commonly used statistical method to express this distribution^53,58^, is based on the “weakest-link” concept^59^. Thus, it assumes that the rupture of the weakest flaw under a given tensile strength initiates the aggregate failure. The survival probability, $P_{s} \left( \sigma \right) ( -$), has been empirically related to the tensile strength, $\sigma$^58^:

3 $$ln\left[ {ln\left( {\frac{1}{{P_{s} }}} \right)} \right] = mln\left( {\frac{\sigma }{{\sigma_{o} }}} \right)$$

where $m \left[ - \right]$ is the slope of the distribution (i.e., referred to as Weibull modulus) characterizing the variability of $\sigma$ and $\sigma_{o} \left[ {Pa} \right]$ is the characteristic tensile strength (where $P_{s}$ corresponds to the 63rd percentile of the Weibull distribution). The values of $\sigma_{o}$ were used to calculate the soil friability, $b \left( - \right)$^21^ as:

4 $$\sigma_{o} = aV^{ - b}$$

where $V \left[ {{\text{m}}^{3} } \right]$ is the average clod volume within a size class and $a \left[ {Pa} \right]$ is an extrapolated estimate of the tensile strength of 1 m^3^ samples of the bulk soil.

### Water content

The mass of the fifteen aggregates in each petri dish was measured immediately before carrying out the tensile strength measurement. The indirect tension tests for tensile strength determination were performed over an aluminum plate and the crushed parts were collected in the petri dish, which was dried in an oven at 105 °C for 24 h. The oven dried aggregate mass was subtracted from the wet mass to calculate the gravimetric water content for each aggregate size class, block and treatment.

### Bulk density and fractal dimension

For bulk density measurements, 10 aggregates from each size class, blocks, soil texture and treatment (total 480 aggregates) were selected and the measurements carried out using the pycnometer GeoPyc 1360 (Micrometrics, Norcross, Georgia, USA)^55^.

The fractal dimension, *D*_*r*_ [−] (0 < *D*_*r*_ < 3), was calculated as^22^:

5 $$\frac{{\rho_{i} }}{{\rho_{u} }} = \frac{{d_{i} }}{{d_{u} }}^{{D_{r} - 3}}$$

where *i* is the rank (in descending order with aggregate size), *u* refers to the largest aggregate and $d \left[ m \right]$ is the diameter of an individual aggregate, calculated by Eq. 2.

### Amorphous silica extraction method

Extraction of silicon (Si) from silica sources was carried out using 0.1 M Tiron (4,5-dihydroxy-1,3-benzene-disulfonic acid (disodium salt), C_6_H_4_Na_2_O_8_S_2_; Roth, Karlsruhe, Germany) as extractant^60,61^. Briefly, 30 mg of soil sample were weighed into 50 mL centrifuge tubes. Subsequently a 30 mL aliquot of 0.1 M Tiron solution was added, followed by heating at 85 °C for 1 h. Prior to and after 30 min of heating, the samples were gently shaken by hand. Finally, the samples were centrifuged at 4000 rpm (5000 g-force) for five minutes and filtered through a 0.45 µm membrane filter. Silicon contents were measured using inductively coupled plasma optical emission spectrometry (ICP-OES; ICP-iCAP 6300 DUO, Thermo scientific, Germany). Samples from every size class, soil texture and treatment were analysed.

### Total carbon content

Total organic (TOC) and inorganic carbon (TIC) contents of the soil were determined by elemental analysis (dry combustion) following DIN ISO 10694^53^ using a Leco RC.612 (Leco instruments GmbH, Germany). Contents were analysed for every size class, soil texture and treatment.

### Statistical analyses

All statistical analyses were carried out in the R software package^54^. To assess variations among treatments in empirical measurements of aggregate characteristics such as tensile strength and Weibull modulus, a two-way analysis of variance was conducted. (ANOVA). Differences between data sets were considered significant at *p* < 0.01 and Tukey’s post hoc test used.

### Random forest resemblance algorithm and variable importance

Data-driven machine learning technique was employed to analyse the importance of each variable on the output. For this, the measured contents of sand, silt, clay, TIC, TOC and silica contents were defined as independent variables. Tensile strength, water content and bulk density were defined as outputs (dependent variables). A total of 20% of the samples were used to train the multiple output regression Random Forest (RF) ensemble learning algorithm^55^. The trained algorithm was used to predict new data combining variables and output. For the assessment of the regression algorithm^56^ the metrics were: 1) coefficient of determination R2-score, which represents the proportion of the variance in the dependent variable that can be explained by the independent variables in the mode (the best possible score is 1.0) and 2) Mean Absolute Error in percentage (MAEp), which finds all absolute errors (xi–x), adds them all and divide by the number of errors. In RF, the depth of a variable used as a decision node in a tree can be used to assess the relative importance of that variable in predicting the output. For this calculation, whose values are positive and sum up to 1.0, the following applies: the higher the value, the more important the contribution of the variable to the prediction function. The mean decrease impurity (MDI) method, available in scikit-learn^62^, was applied to rank the numerical features by their importance for each output using python. This method assesses the impact of each feature on reducing impurity in decision trees, providing valuable insights into feature importance.

The SHAP algorithm (SHapley Additive exPlanations)^63^ was utilized to interpret variable importance in detail. This algorithm employs game theory concepts, such as Shapley values, to clarify the outcomes of tree-based models. It enhances interpretability by developing a rapid algorithm for computing optimal explanations using game theory, introducing a novel explanation type that illustrates local feature interactions and offering new tools to comprehend the overall model structure through the combination of multiple explanations for each prediction.

### Supplementary Information

Supplementary Figure 1.

Supplementary Figure 2.

Supplementary Figure 3.

Supplementary Figure 4.

Supplementary Figure 5.

Supplementary Figure 6.

Supplementary Figure 7.

Supplementary Figure 8.

Supplementary Figure 9.

## Data Availability

The datasets used and/or analyzed during the current study available from the corresponding author on reasonable request.
