# Supplementary material for: Synergistic effects of organic carbon and silica in preserving structural stability of drying soils
Source: Sci Rep. 2024 Apr 9;14:8330. doi: 10.1038/s41598-024-58916-9 (PMC11004191; doi:10.1038/s41598-024-58916-9)
Supplement: Supplementary file 1 — Supplementary Figure 1. [file 41598_2024_58916_MOESM1_ESM.docx]

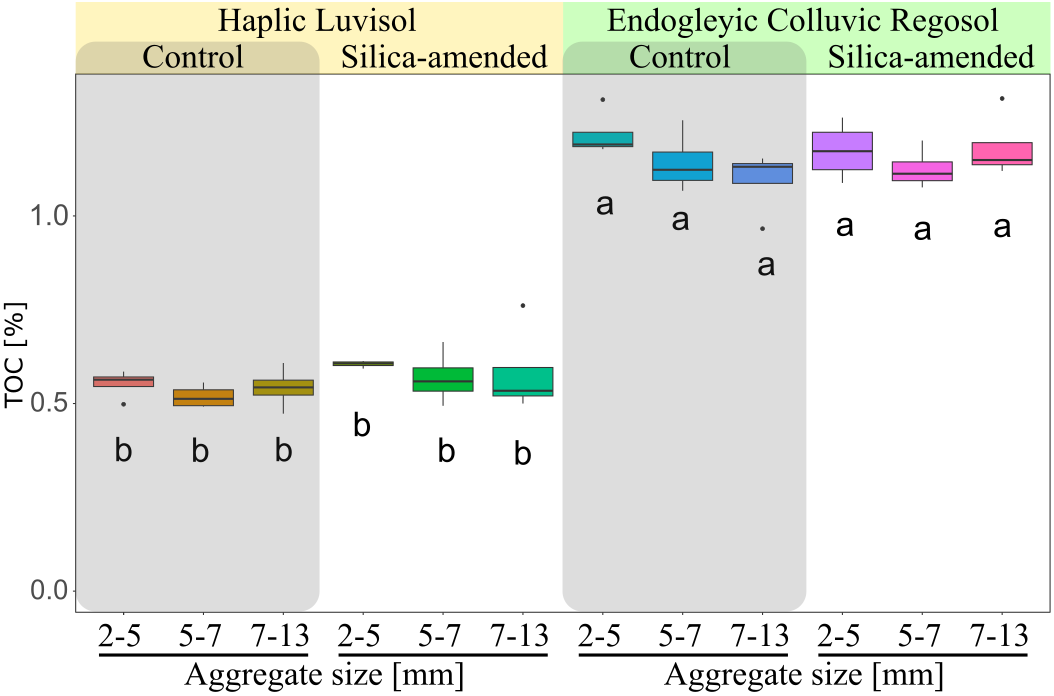


Supplementary material SF.1) Total organic carbon for the soils and treatments used in the study. Different letters indicate statistical significant difference (p<0.05). Boxplot starts in the first quartile (25%) and ends in the third (75%), with a line inside that represents the median. The segment on each side of the box is drawn to the minimum and maximum without counting outliers, that are represented with black points.
