# Supplementary material for: Synergistic effects of organic carbon and silica in preserving structural stability of drying soils
Source: Sci Rep. 2024 Apr 9;14:8330. doi: 10.1038/s41598-024-58916-9 (PMC11004191; doi:10.1038/s41598-024-58916-9)
Supplement: Supplementary file 4 — Supplementary Figure 4. [file 41598_2024_58916_MOESM4_ESM.docx]

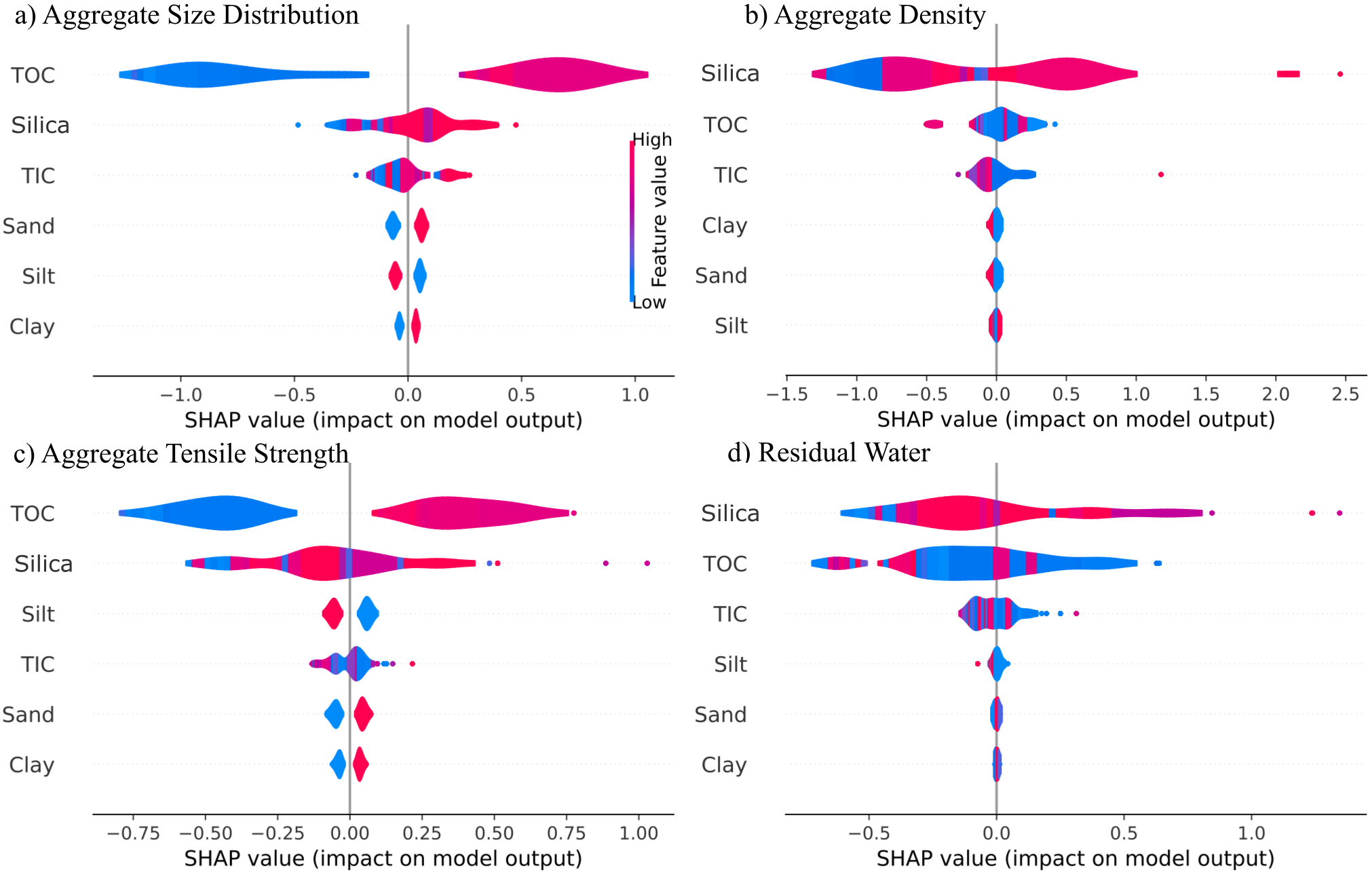


Supplementary material SF.4) SHAP algorithm (SHapley Additive exPlanations) explaining the trained random forest algorithm. Warmer colors signify a positive effect (high feature value) of the feature on the dependent variable, whereas cooler colors indicate a negative effect. TOC and TIC represents the total organic carbon and total inorganic carbon for the soils and treatments utilized in the study, respectively.
