# Supplementary material for: Synergistic effects of organic carbon and silica in preserving structural stability of drying soils
Source: Sci Rep. 2024 Apr 9;14:8330. doi: 10.1038/s41598-024-58916-9 (PMC11004191; doi:10.1038/s41598-024-58916-9)
Supplement: Supplementary file 7 — Supplementary Figure 7. [file 41598_2024_58916_MOESM7_ESM.docx]

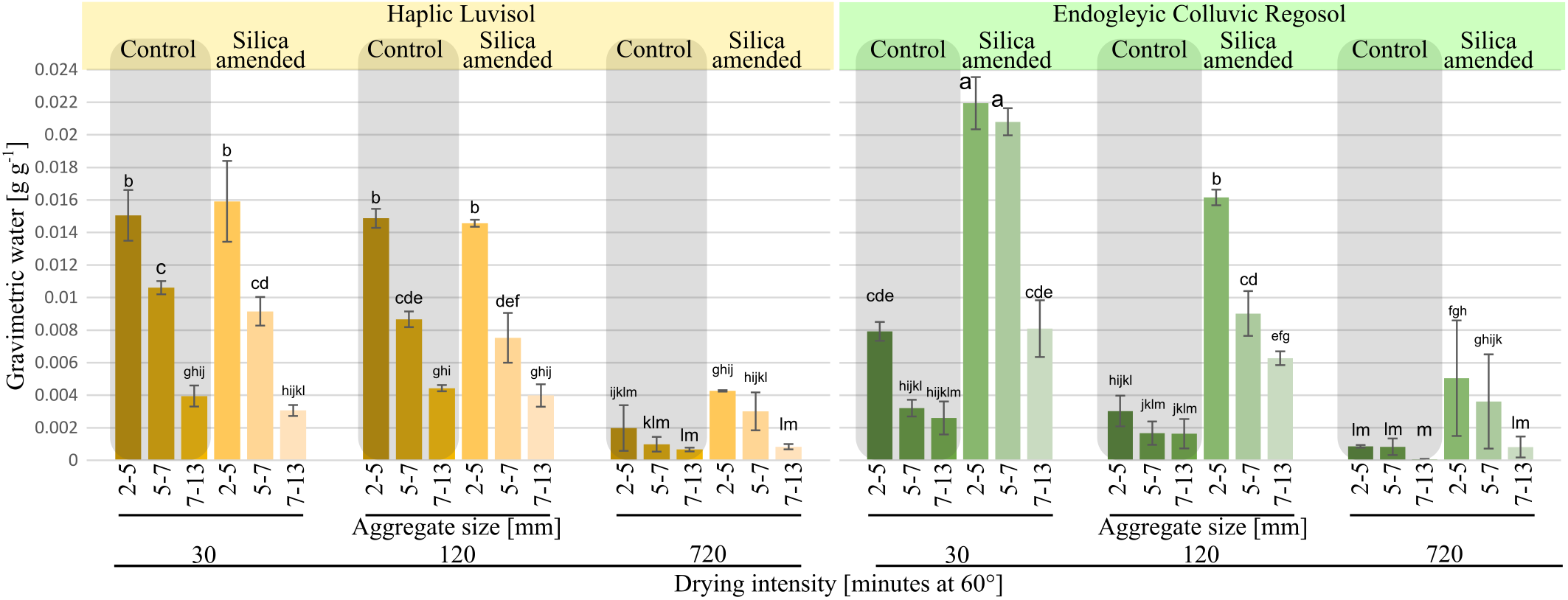


Supplementary material SF.7) Gravimetric water content for each soil and treatment used in the study. Different letters indicate statistical significant difference (p<0.05). The vertical segments indicate standard errors.
